# Daily exercise improves the long-term prognosis of patients with acute coronary syndrome

**DOI:** 10.3389/fpubh.2023.1126413

**Published:** 2023-03-15

**Authors:** Qiang Hu, Peng-Xiao Li, Yu-Shan Li, Qiang Ren, Jian Zhang, Yan-Chun Liang, Quan-Yu Zhang, Ya-Ling Han

**Affiliations:** ^1^Department of Cardiology, General Hospital of Northern Theater Command, Shenyang, China; ^2^Department of Cardiology, Air Force Hospital of Western Theater Command, Chengdu, China; ^3^Department of Cardiology, Xijing Hospital, Air Force Medical University, Xi'an, Shaanxi, China

**Keywords:** nomogram, cardiac rehabilitation, exercise, MACE, education

## Abstract

**Objective:**

To demonstrate the effect of daily exercise on the incidence of major adverse cardiovascular events (MACE) for patients with acute coronary syndrome (ACS).

**Methods:**

A cohort of 9,636 patients with ACS were consecutively enrolled in our retrospective study between November 2015 and September 2017, which were used for model development. 6,745 patients were assigned as the derivation cohort and 2,891 patients were assigned as the validation cohort. The least absolute shrinkage and selection operator (LASSO) regression and COX regression were used to screen out significant variables for the construction of the nomogram. Multivariable COX regression analysis was employed for the development of a model represented by a nomogram. The nomogram was then evaluated for performance traits such as discrimination, calibration, and clinical efficacy.

**Results:**

Among 9,636 patients with ACS (mean [SD] age, 60.3 [10.4] years; 7,235 men [75.1%]), the 5-year incidence for MACE was 0.19 at a median follow-up of 1,747 (1,160–1,825) days. Derived from the LASSO regression and COX regression, the nomogram has included 15 factors in total including age, previous myocardial infarction (MI), previous percutaneous coronary intervention (PCI), systolic pressure, N-terminal Pro-B-type natriuretic peptide (NT-proBNP), high-density lipoprotein cholesterol (HDL), serum creatinine, left ventricular end-diastolic diameter (LVEDD), Killip class, the Synergy between Percutaneous Coronary Intervention with Taxus and Cardiac Surgery (SYNTAX) score, left anterior descending (LAD) stenosis (≥50%), circumflex (LCX) stenosis (≥50%), right coronary artery (RCA) stenosis (≥50%), exercise intensity, cumulative time. The 5-year area under the ROC curve (AUC) of derivation and validation cohorts were 0.659 (0.643–0.676) and 0.653 (0.629–0.677), respectively. The calibration plots showed the strong concordance performance of the nomogram model in both two cohorts. Moreover, decision curve analysis (DCA) also showed the usefulness of nomogram in clinical practice.

**Conclusion:**

The present work provided a prediction nomogram predicting MACE for patients with ACS after incorporating the already known factors and the daily exercise, which demonstrated the effectiveness of daily exercise on the improvement of prognosis for patients with ACS.

## Introduction

Coronary heart disease (CHD), with a high prevalence of 7.2% in US adults is a highly fatal disease, which results in myocardial ischemia and hypoxia (1) and it has also been the second leading disease in increasing the percentage of disability-adjusted life-years (DALYs) (2). According to recent studies (3), the total number of deaths from CHD has risen steadily to 9.14 million in 2019. While in China, the number of death resulting from CHD was about 1 million people, which has grown over the last 20 years, becoming the second leading cause of death (4). As a result, CHD is a major public health problem, thus, its diagnosis and prognosis require particular attention.

To our knowledge, several previous studies have selected and analyzed the potential risk factors related to the high incidence of cardiovascular outcomes in patients with CHD (5–14). GRACE and Thrombolysis in Myocardial Infarction (TIMI) risk scores were recommended to guide clinical practice for patients with ACS in the short or medium term (9, 10). As for patients with acute myocardial infarction (AMI), the PAMI and CADILLAC risk scores were both used to predict 30-day and 1-year mortality in patients after percutaneous coronary intervention (PCI) (8, 11). However, these risk scores were limited by the short follow-up and were not accurately reflect the prognoses of patients with CHD, especially in the Chinese population (6, 12, 13).

Exercise, as an important part of cardiac rehabilitation (CR), plays a pivotal role in the recovery of exercise capacity and the improvement of quality of life. Exercise-based cardiac rehabilitation could not only improve exercise capacity and quality of life but also reduce the rates of mortality and myocardial infarction (15–22). Therefore, exercise is effective for reducing major adverse cardiovascular events (MACE) and imposes a positive effect on the prognoses of patients with CHD. However, few studies have yet designed a nomogram combining clinical indicators and regular exercise to predict the risk of patients with CHD. In our study, we selected patients with ACS in China as our targeted population to develop and validate a long-term prognostic nomogram incorporating daily exercise in order to help high-risk patient identification and clinical decision-making.

## Materials and methods

### Patient selection and study design

**Figure 1:**
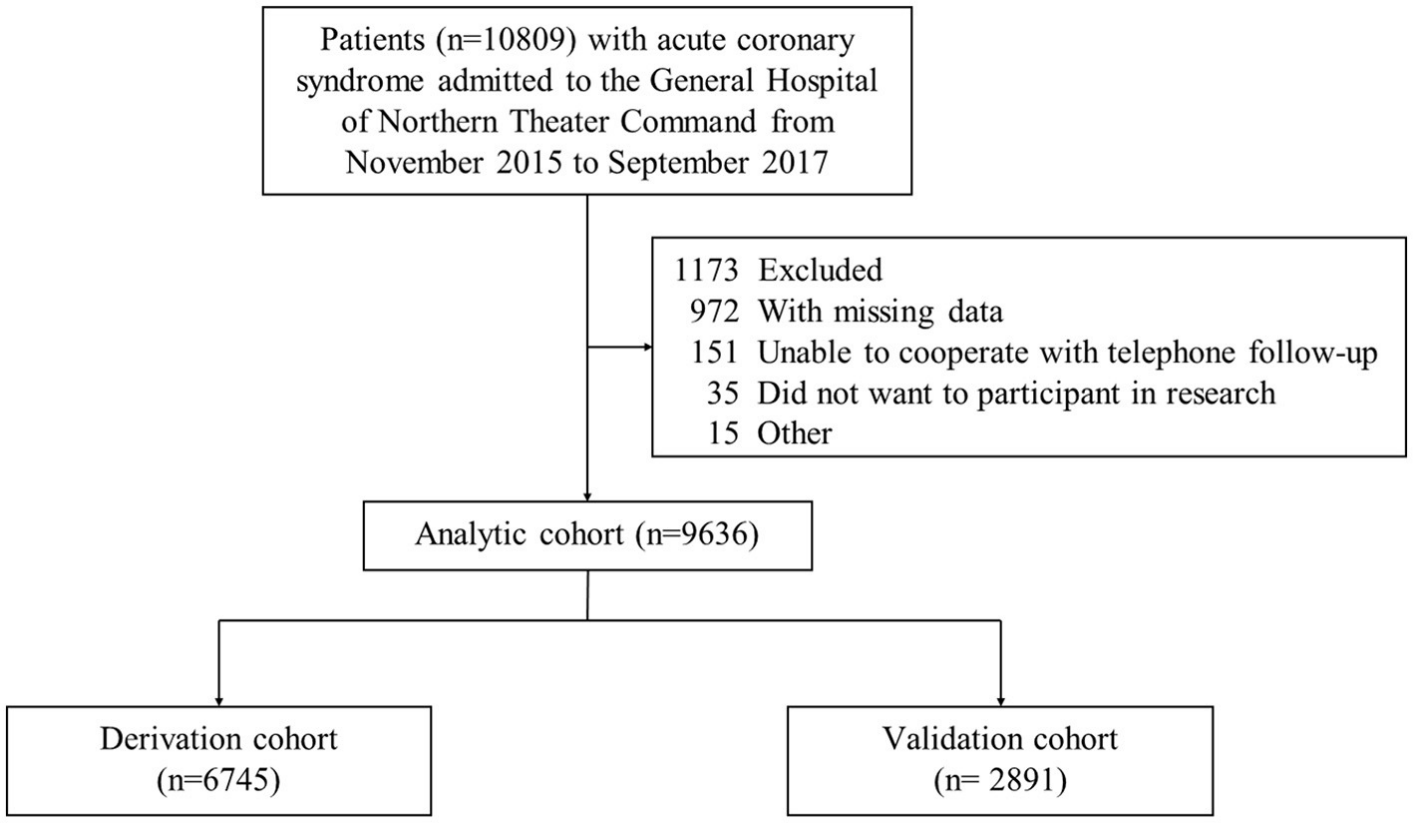
Study flow diagram.

A total of 10,809 patients with ACS were retrospectively enrolled at our hospital between November 2015 and September 2017. Inclusion criteria were as follows: (i) age over 18 years old; (ii) patients diagnosed as ACS (23, 24); (iii) patients receiving coronary angiography. Exclusion criteria were as follows: (i) incomplete information; (ii) unable to cooperate with the telephone follow-up. Based on the acceptance criteria, 1,173 patients were excluded and 9,636 patients with ACS were eligible for our study (Figure 1). Of those, 6,745 patients were assigned as the derivation cohort and 2,891 patients were assigned as the validation cohort according to the random sequence generated by the computer.

Our study is an open-label, retrospective trial. Patients who had a MACE record during the follow-up were defined as the MACE group, whereas the patients without MACE were assigned as the control group. The Institutional Ethics Committee of the General Hospital of Northern Theater Command approved our study and waived the need for patient consent. The study was conducted in accordance with the Declaration of Helsinki.

### Clinical data collection

Based on the clinical experience and previous studies (8–11), 50 candidate variables were identified for establishing the model. Potential predictors include the following characteristics of the patient: demographic characteristics [e.g., age, gender, smoking status, drinking status, and body mass index (BMI)], medical history [e.g., hypertension, diabetes, stroke, previous myocardial infarction (MI), previous PCI, and chronic kidney disease], medication history [e.g., aspirin, clopidogrel, angiotensin-converting enzyme inhibitors (ACEI), and angiotensin receptor blockers (ARB)], cardiac ultrasonography [e.g., left ventricular end-diastolic diameter (LVEDD) and left ventricular ejection fraction (LVEF)], laboratory parameters [e.g., hemoglobin, blood lipids, serum creatinine and N-terminal Pro-B-type natriuretic peptide (NT-proBNP)], surgery-related indices[three-vessel disease, arterial access site, the Synergy between Percutaneous Coronary Intervention with Taxus and Cardiac Surgery (SYNTAX) score, intra-aortic balloon counterpulsation (IABP), number of stents, and chronic total occlusion (CTO)] and exercise-related indicators (exercise intensity, cumulative time, and exercise type). All the data were recorded at the admission of patients and collected through the cv net clinical data collection system (Beijing Crealife Technology Co., Ltd.).

### Outcomes and follow-up

The primary outcome was a 5-year MACE, which was the composite endpoint of all-cause death, stroke, MI, and target vessel revascularization (TVR). 1- and 3-year MACE were also assessed. The follow-up was performed in 1, 3, 6, 12, 24, 36, 48, and 60 months to record the outcome, until the patient's first MACE occurred or the study was terminated by the follow-up on August 31, 2022. The exercise-related indicators were also collected during the follow-up (25). The exercise intensity was categorized as mild (< 3 METs), moderate (3–6 METs), and high intensity (>6 METs) (Supplementary eTable 1). The exercise type comprised walking, riding, running, and swimming. The cumulative time was defined as the cumulative time of walking, light household activities, and exercise in a week. The exercise intensity and cumulative time were defined as the average level at the 5-year follow-up. The follow-up was conducted by the follow-up staff via telephone interview.

### Construction and validation of nomogram

As shown in Supplementary eFigure 1, the entire data set was randomly divided into the derivation cohort and validation cohort in a ratio of 7:3. All variable names in our study were encoded to V1–V50 to achieve statistical blindness. In the derivation cohort, the significance of each variable was examined using the least absolute shrinkage and selection operator (LASSO) regression and univariable Cox regression to screen out the independent risk factors that affected the prognosis of patients with ACS. Variables with significant differences (*P* < 0.05) were taken as the candidate variables and included in the multivariable COX proportional hazards regression model. Optimization of the model was further performed using the stepwise method (Akaike information criterion). Before the construction of the final model, each encoded variable names were displayed as variable names. The nomogram was developed from the variables that had *P*-values < 0.05 in the final model.

The performance evaluation of the nomogram included discrimination, calibration, and clinical efficacy. The area under the time-dependent receiver operating characteristic curves was used to evaluate the discrimination capacity in both the derivation cohort and validation cohort. The discrimination of the nomogram was further verified using the Harrel concordance index. The degree of the calibration was determined by the calibration plot. The decision curve analysis (DCA) was performed to evaluate the clinical efficacy of the nomogram.

### Statistical analysis

Statistical analysis was performed using R software, version 4.2.0 (R Foundation for Statistical Computing). The categorical variables were expressed as the case number and percentage (%) and the chi-square test or Fisher's exact test was used for statistical comparison between groups. The normal distribution test was performed for continuous variables. Normally distributed variables were presented as the mean ± standard deviation (mean ± SD). The comparison between the two groups was conducted by independent sample *t*-test. Those variables without normal distribution were summarized as the medians and interquartile ranges (IQR) and compared by the Mann–Whitney *U*-test. Moreover, exercise volume was defined as the product of exercise intensity and cumulative time. The restricted cubic spline model fitted for the COX proportional hazard model with 4 knots at the 5^th^, 35^th^, 65^th^, and 95^th^ percentiles of exercise volume was then used to determine the diagnostic threshold for patients. Patients with higher or lower exercise volume were classified into the high exercise volume group or the low exercise volume group in the full cohort. The Kaplan–Meier (KM) curve was then drawn for survival analysis according to different exercise volumes. In addition, variables with missing data < 20% were imputed by multiple imputation. All statistical tests were two-tailed and a *P* < 0.05 was considered statistically significant.

## Results

### Clinical features and characteristics

A total of 9,636 patients with ACS were eligible for our study. The mean (SD) age was 60.33 (10.36) years and 7,235 patients (75.08%) were male (Table 1). Among all patients, 6,745 patients were assigned to the derivation cohort and 2,891 patients were assigned to the validation cohort. There was no significant difference between the derivation and validation cohort (all *P* > 0.05) (Supplementary eTable 2).

**Table 1:** Baseline demographics and clinical characteristics of MACE in patients with ACS.

**Characteristic**	**Overall (*n* = 9,636)**	**MACE (*n* = 1,791)**	**Control (*n* = 7,845)**	***P***
Age (years)	60.33 ± 10.36	62.43 ± 10.66	59.86 ± 10.24	< 0.001
Male, no. (%)	7,235 (75.08%)	1,355 (75.66%)	5,880 (74.95%)	0.534
Body mass index (kg/m^2^)	24.34 ± 3.10	24.46 ± 3.10	24.31 ± 3.10	0.063
**Smoking status, no. (%)**				
No smoke	4,036 (41.88%)	716 (39.98%)	3,320 (42.32%)	0.193
Current smoker	4,175 (43.33%)	802 (44.78%)	3,373 (43.00%)	
Previous smoker	1,425 (14.79%)	273 (15.24%)	1,152 (14.68%)	
**Drinking status, no. (%)**				
No drink	7,209 (74.81%)	1,346 (75.15%)	5,863 (74.74%)	0.006
Current drinker	1,908 (19.80%)	325 (18.15%)	1,583 (20.18%)	
Previous drinker	519 (5.39%)	120 (6.70%)	399 (5.09%)	
**Diagnosis, no. (%)**				
UA	6,548 (67.95%)	1,183 (66.05%)	5,365 (68.39%)	0.161
NSTEMI	1,282 (13.30%)	252 (14.07%)	1,030 (13.13%)	
STEMI	1,806 (18.74%)	356 (19.88%)	1,450 (18.48%)	
Hypertension, no. (%)	6,155 (63.88%)	1,193 (66.61%)	4,962 (63.25%)	0.008
Previous MI, no. (%)	1,742 (18.08%)	401 (22.39%)	1,341 (17.09%)	< 0.001
Chronic kidney disease, no. (%)	95 (0.99%)	30 (1.68%)	65 (0.83%)	0.001
Diabetes, no. (%)	2,569 (26.66%)	560 (31.27%)	2,009 (25.61%)	< 0.001
Previous PCI, no. (%)	1,813 (18.81%)	419 (23.39%)	1,394 (17.77%)	< 0.001
Stroke, no. (%)	443 (4.60%)	116 (6.48%)	327 (4.17%)	< 0.001
Heart rate (beats/min)	76.71 ± 13.60	77.34 ± 14.59	76.57 ± 13.36	0.137
Systolic pressure (mmHg)	135.75 ± 21.40	137.00 ± 23.07	135.46 ± 21.00	0.004
Diastolic pressure (mmHg)	78.34 ± 12.56	78.18 ± 13.13	78.37 ± 12.42	0.702
NT-proBNP (pg/mL)	203.40 (69.92, 738.62)	320.30 (92.08, 1,135.00)	185.50 (65.81, 663.20)	< 0.001
Triglyceride (mmol/L)	1.84 ± 1.49	1.85 ± 1.45	1.84 ± 1.50	0.615
LDL (mmol/L)	2.36 ± 0.81	2.38 ± 0.84	2.35 ± 0.80	0.382
HDL (mmol/L)	0.92 ± 0.21	0.91 ± 0.21	0.92 ± 0.21	0.002
Cholesterol (mmol/L)	4.16 ± 1.13	4.18 ± 1.16	4.15 ± 1.12	0.760
cTnT (ng/L)	0.02 (0.01, 0.27)	0.03 (0.01, 0.51)	0.02 (0.01, 0.23)	< 0.001
Serum creatinine (umol/L)	70.47 (60.46, 81.67)	72.80 (61.81, 86.82)	70.00 (60.25, 80.69)	< 0.001
CKMB (U/L)	12.00 (10.00, 18.00)	13.00 (10.00, 20.00)	12.00 (10.00, 18.00)	0.022
Hemoglobin (g/L)	136.47 ± 15.20	134.48 ± 16.59	136.92 ± 14.83	< 0.001
LVEDD (mm)	49.69 ± 6.04	50.43 ± 6.34	49.52 ± 5.96	< 0.001
LVEF (%)	58.94 ± 8.50	57.84 ± 9.29	59.19 ± 8.29	< 0.001
**Killip class, no. (%)**				
0	6,488 (67.33%)	1,123 (62.70%)	5,365 (68.39%)	< 0.001
I	2,810 (29.16%)	549 (30.65%)	2,261 (28.82%)	
II	258 (2.68%)	77 (4.30%)	181 (2.31%)	
III	35 (0.36%)	18 (1.01%)	17 (0.22%)	
IV	45 (0.47%)	24 (1.34%)	21 (0.27%)	
IABP, no. (%)	115 (1.19%)	38 (2.12%)	77 (0.98%)	< 0.001
SYNTAX score	14.00 ± 8.96	16.46 ± 9.72	13.43 ± 8.68	< 0.001
Three-vessel disease, no. (%)	3,663 (38.01%)	898 (50.14%)	2,765 (35.25%)	< 0.001
Chronic total occlusion, no. (%)	70 (0.73%)	19 (1.06%)	51 (0.65%)	0.065
**Arterial access site, no. (%)**				
Femoral	589 (6.11%)	157 (8.77%)	432 (5.51%)	< 0.001
Radial	9,047 (93.89%)	1,634 (91.23%)	7,413 (94.49%)	
Contrast (ml)	100.00 (40.00, 180.00)	100.00 (50.00–190.00)	100.00 (40.00, 170.00)	< 0.001
LAD stenosis (≥50%), no. (%)	8,086 (83.91%)	1,578 (88.11%)	6,508 (82.96%)	< 0.001
LCX stenosis (≥50%), no. (%)	5,654 (58.68%)	1,253 (69.96%)	4,401 (56.10%)	< 0.001
RCA stenosis (≥50%), no. (%)	6,171 (64.04%)	1,341 (74.87%)	4,830 (61.57%)	< 0.001
Number of stents	1.39 ± 0.88	1.51 ± 0.83	1.36 ± 0.88	< 0.001
Aspirin, no. (%)	9,531 (98.91%)	1,767 (98.66%)	7,764 (98.97%)	0.258
Clopidogrel, no. (%)	8,447 (87.66%)	1,583 (88.39%)	6,864 (87.50%)	0.301
Ticagrelor, no. (%)	2,353 (24.42%)	416 (23.23%)	1,937 (24.69%)	0.193
Statin, no. (%)	9,378 (97.32%)	1,748 (97.60%)	7,630 (97.26%)	0.422
Beta blocker, no. (%)	6,865 (71.24%)	1,262 (70.46%)	5,603 (71.42%)	0.419
Calcium channel blocker, no. (%)	2,727 (28.30%)	530 (29.59%)	2,197 (28.01%)	0.178
Nitrate, no. (%)	6,291 (65.29%)	1,189 (66.39%)	5,102 (65.04%)	0.278
Proton-pump inhibitor, no. (%)	6,426 (66.69%)	1,220 (68.12%)	5,206 (66.36%)	0.154
ACEI/ARB, no. (%)	5,760 (59.78%)	1,081 (60.36%)	4,679 (59.64%)	0.578
Diuretics, no. (%)	1,633 (16.95%)	421 (23.51%)	1,212 (15.45%)	< 0.001
**Exercise intensity, no. (%)** ^a^				
Mild	1,015 (10.53%)	226 (12.62%)	789 (10.06%)	< 0.001
Moderate	8,580 (89.04%)	1,563 (87.27%)	7,017 (89.45%)	
High	41 (0.43%)	2 (0.11%)	39 (0.50%)	
Cumulative time (h/week)^b^	3.00 (2.00,6.00)	3.00 (2.00,5.00)	3.00 (2.00,6.00)	< 0.001
**Exercise type, no. (%)**				
No exercise	32 (0.33%)	7 (0.39%)	25 (0.32%)	0.838
Walking	9,571 (99.33%)	1,780 (99.39%)	7,791 (99.31%)	
Riding	9 (0.09%)	1 (0.06%)	8 (0.10%)	
Running or Swimming	24 (0.25%)	3 (0.17%)	21 (0.27%)	

MACE, major adverse cardiovascular events; ACS, acute coronary syndrome; UA, unstable angina; NSTEMI, non-ST segment elevation myocardial infarction; STEMI, ST-segment elevation myocardial infarction; MI, myocardial infarction; PCI, percutaneous coronary intervention; NT-proBNP, N-terminal Pro-B-type natriuretic peptide; LDL, low density lipoprotein cholesterol; HDL, high density lipoprotein cholesterol; cTnT, troponinT; CKMB, creatine kinase isoenzyme; LVEDD, left ventricular end-diastolic diameter; LVEF, left ventricular ejection fraction; IABP, intra-aortic balloon counterpulsation; SYNTAX score, the synergy between percutaneous coronary intervention with taxus and cardiac surgery score; LAD, left anterior descending; LCX, circumflex; RCA, right coronary artery; ACEI, angiotensin converting enzyme inhibitors; ARB, angiotensin receptor blockers.

^a^Physical activity was categorized into mild (< 3 METs), moderate (3–6 METs) and high intensity (>6 METs).

^b^Cumulative exercise time was defined as the cumulative time of walking, light household activities, and exercise in a week.

The median follow-up time was 1,747 (1,160–1,825) days. The incidences of MACE in the derivation cohort were 0.05, 0.14, and 0.19, respectively after 1, 3, and 5 years. For the validation cohort, after 1, 3, and 5 years, MACE rates were 0.06, 0.14, and 0.19, respectively. Comparisons between the two cohorts showed no significant differences (*P* > 0.05). The MACE group has the following characteristics in comparison with the control group: older age, drinking status, hypertension, previous MI, chronic kidney disease, diabetes, previous PCI, stroke, systolic pressure, NT-proBNP, HDL, troponin T (cTnT), serum creatinine, creatine kinase isoenzyme (CKMB), hemoglobin, LVEDD, LVEF, Killip class, IABP, SYNTAX score, three-vessel disease, arterial access site, contrast, left anterior descending (LAD) stenosis (≥50%), circumflex (LCX) stenosis (≥50%), right coronary artery (RCA) stenosis (≥50%), number of stents, diuretics, exercise intensity, cumulative time (Table 1, all *P* < 0.05).

### Predictive nomogram construction

**Figure 2:**
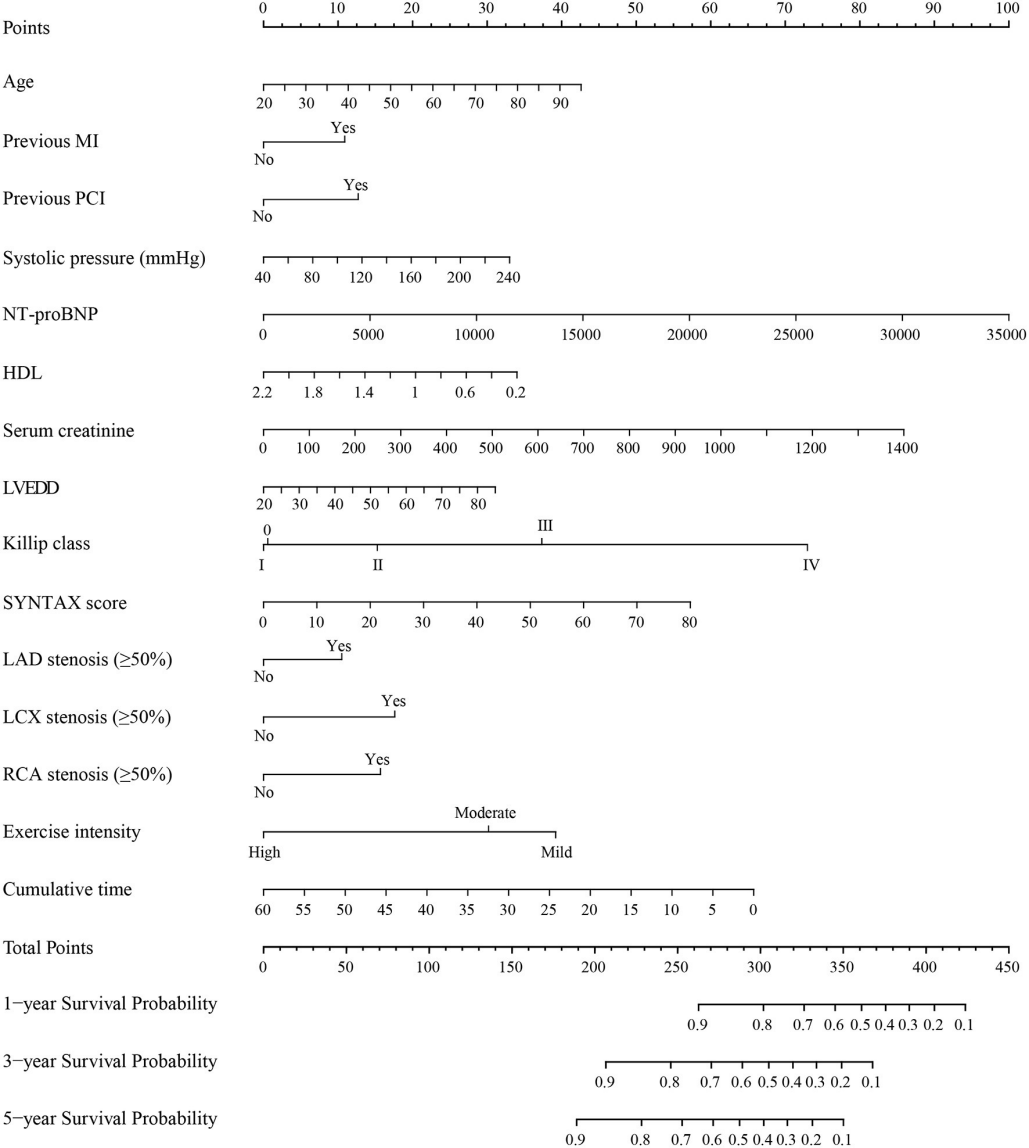
Nomogram for predicting MACE in patients with ACS. MACE, major adverse cardiovascular events; ACS, acute coronary syndrome; MI, myocardial infarction; PCI, percutaneous coronary intervention; NT-proBNP, N-terminal Pro-B-type natriuretic peptide; HDL, high density lipoprotein cholesterol; LVEDD, left ventricular end-diastolic diameter; SYNTAX score, the synergy between percutaneous coronary intervention with taxus and cardiac surgery score; LAD, left anterior descending; LCX, circumflex; RCA, right coronary artery. Instructions for use of nomogram: the score of each variable was obtained by drawing a vertical line upward to the points line, then the sum of these scores was plotted on the total points line. Finally, the probability of 1-, 3-, and 5-year MACE was determined by drawing the vertical line according to the total points until it intersected with each survival axis.

After 50 candidate variables entered the LASSO regression, 39 clinical variables were found to meet the threshold of *P* < 0.05 (Supplementary eFigures 2A, B). In the univariable COX regression analysis, 27 variables were significantly associated with the incidence of MACE (Table 2). The multivariable COX regression analysis demonstrated that age [hazard ratio (HR), 1.01; 95%CI, 1.00–1.02; *P* = 0.003], previous MI (HR, 1.24; 95%CI, 1.07–1.43; *P* = 0.005), previous PCI (HR, 1.27; 95%CI, 1.10–1.47; *P* = 0.001), systolic pressure (HR, 1.00; 95%CI, 1.00–1.01; *P* = 0.013), NT-proBNP (HR, 1.00; 95%CI, 1.00–1.00; *P* < 0.001), HDL (HR, 0.71; 95%CI, 0.53–0.94; *P* = 0.015), serum creatinine (HR 1.00; 95%CI, 1.00–1.00; *P* = 0.059), LVEDD (HR, 1.01; 95%CI, 1.00–1.02; *P* = 0.043), Killip class (I: HR, 2.45; 95%CI, 1.61–3.73; II: HR, 1.59; 95%CI, 1.12–2.26; III: HR, 1.12; 95%CI, 0.72–1.75; IV: HR: 1.13; 95%CI, 0.78–1.65; *P* = 0.001), SYNTAX score (HR, 1.01; 95%CI, 1.00–1.02; *P* = 0.001), LAD stenosis (HR, 1.22; 95%CI, 1.01–1.48; *P* = 0.036), LCX stenosis (HR, 1.39; 95%CI, 1.22–1.59; *P* < 0.001), RCA stenosis (HR, 1.35; 95%CI, 1.18–1.55; *P* < 0.001), and cumulative time (HR, 0.98; 95%CI, 0.97–1.00; *P* = 0.023) were significant independent predictors of the MACE rate for patients with ACS (Table 2). No variables were removed when using the stepwise method (Akaike information criterion) and then these predictors and exercise intensity of interest were used to construct the prognostic risk model (Table 3). After drawing a vertical line from the corresponding axis of each predictor until it reaches the point axis, scores for corresponding variables were obtained, summed, and located on the “Total Points” axis. Then, another vertical line descending from the axis was drawn until it intersects the probability axis to determine the 1-, 3-, and 5-year probabilities of MACE (Figure 2).

**Table 2:** Univariate and multivariable analysis of MACE in patients with ACS.

**Characteristic**	**Univariate**		**Multivariable**	
	**analysis**		**analysis**	
	**HR (95%** ***CI*****)**	***P***	**HR (95%** ***CI*****)**	***P***
Age	1.02 (1.02–1.03)	< 0.001	1.01 (1.00–1.02)	0.003
Male	1.06 (0.93–1.21)	0.347		
Body mass index	1.01 (0.99–1.03)	0.183		
Smoking status		0.365		
No smoke	Ref			
Current smoker	1.06 (0.94–1.19)			
Previous smoker	1.12 (0.95–1.32)			
Drinking status		0.071		
No drink	Ref			
Current drinker	0.91 (0.79–1.04)			
Previous drinker	1.22 (0.97–1.52)			
Diagnosis		0.897		
UA	Ref			
NSTEMI	1.01 (0.85–1.19)			
STEMI	1.03 (0.90–1.20)			
Previous MI	1.47 (1.29–1.67)	< 0.001	1.24 (1.07–1.43)	0.005
Diabetes	1.27 (1.13–1.43)	< 0.001	1.05 (0.92–1.18)	0.5
Previous PCI	1.38 (1.21–1.57)	< 0.001	1.27 (1.10–1.47)	0.001
Stroke	1.46 (1.15–1.85)	0.003	1.26 (0.99–1.60)	0.064
Heart rate	1.00 (1.00–1.01)	0.029	1.00 (1.00–1.01)	0.6
Systolic pressure	1.00 (1.00–1.01)	0.005	1.00 (1.00–1.01)	0.013
NT-proBNP	1.00 (1.00–1.00)	< 0.001	1.00 (1.00–1.00)	< 0.001
LDL	1.05 (0.98–1.13)	0.139		
HDL	0.72 (0.55–0.95)	0.018	0.71 (0.53–0.94)	0.015
cTnT	1.05 (1.03–1.09)	< 0.001	1.02 (0.97–1.06)	0.6
Serum creatinine	1.00 (1.00–1.00)	< 0.001	1.00 (1.00–1.00)	0.059
CKMB	1.00 (1.00–1.00)	0.004	1.00 (1.00–1.00)	0.3
Hemoglobin	0.99 (0.99–1.00)	< 0.001	1.00 (0.99–1.00)	0.4
LVEDD	1.02 (1.01–1.03)	< 0.001	1.01 (1.00–1.02)	0.043
LVEF	0.98 (0.98–0.99)	< 0.001	1.00 (1.00–1.01)	0.4
Killip class		< 0.001		0.001
0	Ref		Ref	
I	3.64 (2.52–5.24)		2.45 (1.61–3.73)	
II	1.15 (0.82–1.62)		1.59 (1.12–2.26)	
III	0.76 (0.50–1.14)		1.12 (0.72–1.75)	
IV	0.94 (0.66–1.33)		1.13 (0.78–1.65)	
IABP	2.42 (1.64–3.56)	< 0.001	1.46 (0.95–2.23)	0.10
SYNTAX score	1.03 (1.03–1.04)	< 0.001	1.01 (1.00–1.02)	0.001
Chronic total occlusion	1.59 (0.96–2.65)	0.095		
Arterial access site		< 0.001		0.5
Femoral	Ref		Ref	
Radial	0.65 (0.53–0.79)		0.92 (0.75–1.14)	
Contrast	1.00 (1.00–1.00)	0.001	1.00 (1.00–1.00)	0.3
LAD stenosis (≥50%)	1.52 (1.28–1.81)	< 0.001	1.22 (1.01–1.48)	0.036
LCX stenosis (≥50%)	1.82 (1.61–2.05)	< 0.001	1.39 (1.22–1.59)	< 0.001
RCA stenosis (≥50%)	1.69 (1.49–1.92)	< 0.001	1.35 (1.18–1.55)	< 0.001
Number of stents	1.18 (1.11–1.25)	< 0.001	1.01 (0.94–1.08)	0.8
Aspirin	0.78 (0.47–1.27)	0.333		
Clopidogrel	1.11 (0.92–1.32)	0.267		
Beta blockers	0.94 (0.83–1.06)	0.323		
Proton-pump inhibitor	1.10 (0.97–1.24)	0.126		
ACEI/ARB	0.98 (0.87–1.10)	0.706		
Diuretics	1.54 (1.35–1.76)	< 0.001	1.16 (0.99–1.35)	0.065
Exercise intensity^a^		0.016		0.12
Mild	Ref		Ref	
Moderate	0.44 (0.16–1.17)		0.59 (0.22–1.59)	
High	0.73 (0.41–1.30)		0.84 (0.47–1.49)	
Cumulative time^b^	0.95 (0.94–0.97)	< 0.001	0.98 (0.97–1.00)	0.023

MACE, major adverse cardiovascular events; ACS: acute coronary syndrome; UA, unstable angina; NSTEMI, non-ST segment elevation myocardial infarction; STEMI, ST-segment elevation myocardial infarction; MI, myocardial infarction; PCI, percutaneous coronary intervention; NT-proBNP, N-terminal Pro-B-type natriuretic peptide; LDL, low density lipoprotein cholesterol; HDL, high density lipoprotein cholesterol; cTnT, troponinT; CKMB, creatine kinase isoenzyme; LVEDD, left ventricular end-diastolic diameter; LVEF, left ventricular ejection fraction; IABP, intra-aortic balloon counterpulsation; SYNTAX score, the synergy between percutaneous coronary intervention with taxus and cardiac surgery score; LAD, left anterior descending; LCX, circumflex; RCA, right coronary artery; ACEI, angiotensin converting enzyme inhibitors; ARB, angiotensin receptor blockers.

^a^Physical activity was categorized into mild (< 3 METs), moderate (3–6 METs) and high intensity (>6 METs).

^b^Cumulative exercise time was defined as the cumulative time of walking, light household activities, and exercise in a week.

**Table 3:** Selected variables for model construction.

**Factors**	**Coefficient**	**HR (95%CI)**	***P***
Age	0.01	1.01 (1.00–1.02)	< 0.001
Previous MI	0.21	1.23 (1.07–1.42)	0.001
Previous PCI	0.24	1.28 (1.11–1.47)	0.001
Systolic pressure	0.00	1.00 (1.00–1.01)	0.020
NT-proBNP	0.00	1.00 (1.00–1.00)	< 0.001
HDL	−0.33	0.72 (0.55–0.95)	0.020
Serum creatinine	0.00	1.00 (1.00–1.00)	0.044
LVEDD	0.01	1.01 (1.00–1.02)	0.053
Killip class			< 0.001
I	1.1	0.99 (0.87–1.12)	
II	0.40	1.33 (0.99–1.77)	
III	−0.01	2.03 (1.09–3.78)	
IV	0.04	4.00 (2.39–6.69)	
SYNTAX score	0.01	1.01 (1.01–1.02)	< 0.001
LAD stenosis (≥50%)	0.20	1.22 (1.01–1.48)	0.007
LCX stenosis (≥50%)	0.34	1.40 (1.23–1.60)	< 0.001
RCA stenosis (≥50%)	0.30	1.35 (1.18–1.55)	< 0.001
Exercise intensity^a^			0.093
Moderate	−0.54	0.84 (0.71–1.00)	
High	−0.17	0.47 (0.11–1.89)	
Cumulative time^b^	−0.02	0.98 (0.96–1.00)	0.008

MI, myocardial infarction; PCI, percutaneous coronary intervention; NT-proBNP, N-terminal Pro-B-type natriuretic peptide; HDL, high density lipoprotein cholesterol; LVEDD, left ventricular end-diastolic diameter; IABP, intra-aortic balloon counterpulsation; SYNTAX score, the synergy between percutaneous coronary intervention with taxus and cardiac surgery score; LAD, left anterior descending; LCX, circumflex; RCA, right coronary artery.

^a^Physical activity was categorized into mild (< 3 METs), moderate (3–6 METs) and high intensity (>6 METs).

^b^Cumulative exercise time was defined as the cumulative time of walking, light household activities, and exercise in a week.

### Validation of nomogram

**Figure 3:**
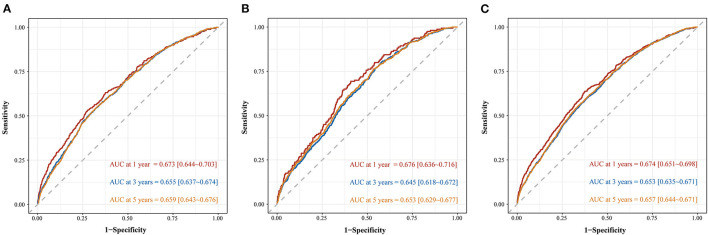
Time-dependent ROC curve of predicting MACE among patients with ACS in derivation cohort **(A)**, validation cohort **(B)**, and full cohort **(C)**.

**Figure 4:**
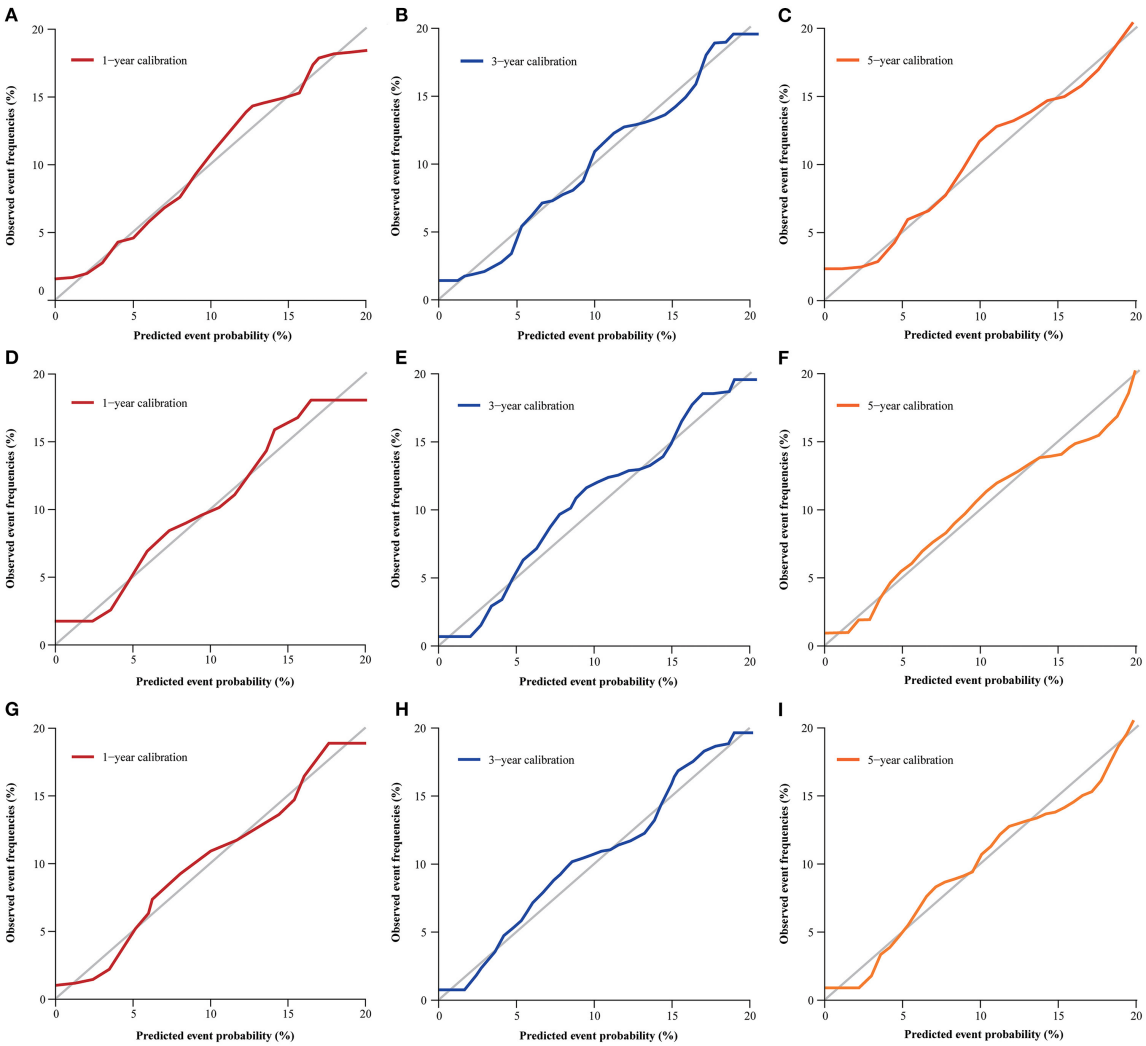
Calibration curve for predicting MACE probability at 1- **(A)**, 3- **(B)**, and 5-year **(C)** in the derivation cohort; 1- **(D)**, 3- **(E)**, and 5-year **(F)** in validation cohort; 1- **(G)**, 3- **(H)**, and 5-year **(I)** in the full cohort.

**Figure 5:**
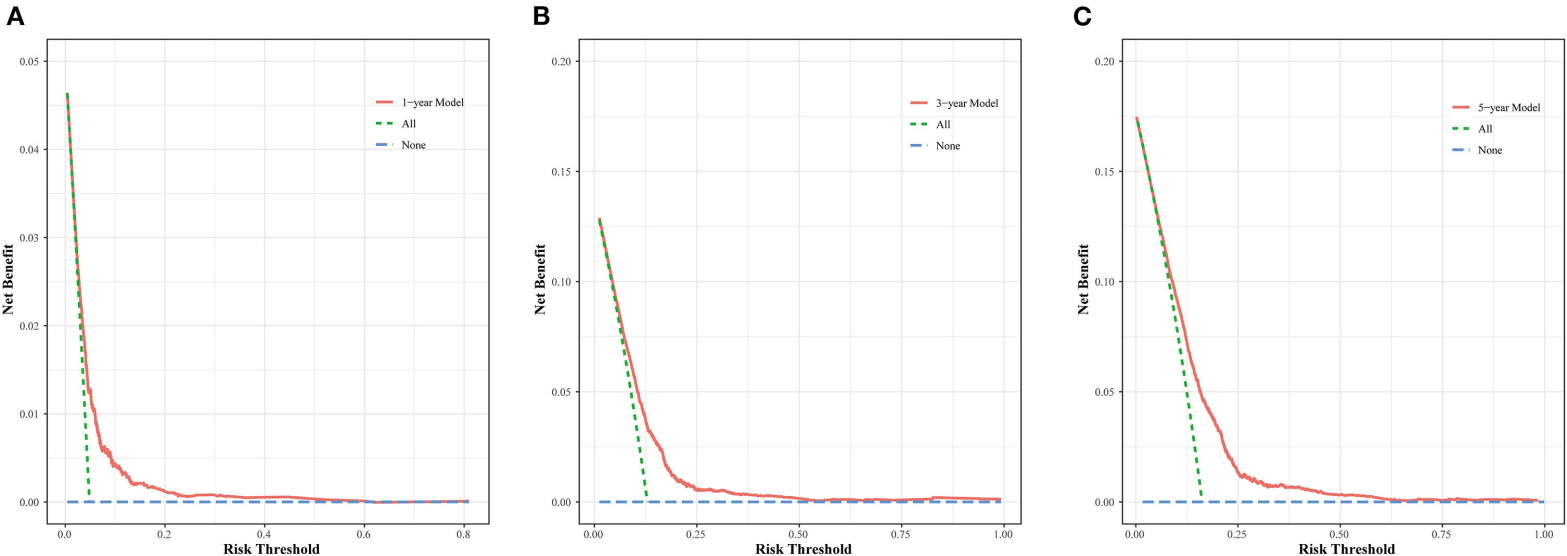
DCA curve for the MACE prediction model at 1- **(A)**, 3- **(B)**, and 5-year **(C)** in the derivation cohort.

The nomogram was evaluated by measuring discrimination, calibration, and clinical efficacy. In the derivation cohort, the 1-year area under the ROC curve (AUC) associated with the MACE was 0.673 (0.644–0.703), the 3-year AUC was 0.655 (0.637–0.674), and the 5-year AUC was 0.659 (0.643–0.676), as shown in Figure 3A. C-index of the nomogram was 0.645 (0.631–0.660). While in the validation cohort, the AUC for predicting MACE at 1, 3, and 5 years were 0.676 (0.636–0.716), 0.645 (0.618–0.672), 0.653 (0.629–0.677), respectively (Figure 3B). The nomogram yielded a C-index of 0.640 (0.618–0.662) for predicting the incidence of MACE, which showed excellent discrimination. The excellent discrimination test for the full cohort was also demonstrated in the Figure 3C. The calibration capacity of the nomogram was assessed by using the calibration curve. Based on the calibration curves, the nomogram showed good agreement for both the derivation and validation cohorts (Figure 4). The DCA of the model in the derivation and validation cohort was presented in Figure 5.

### Exercise volume-based survival analysis

**Figure 6:**
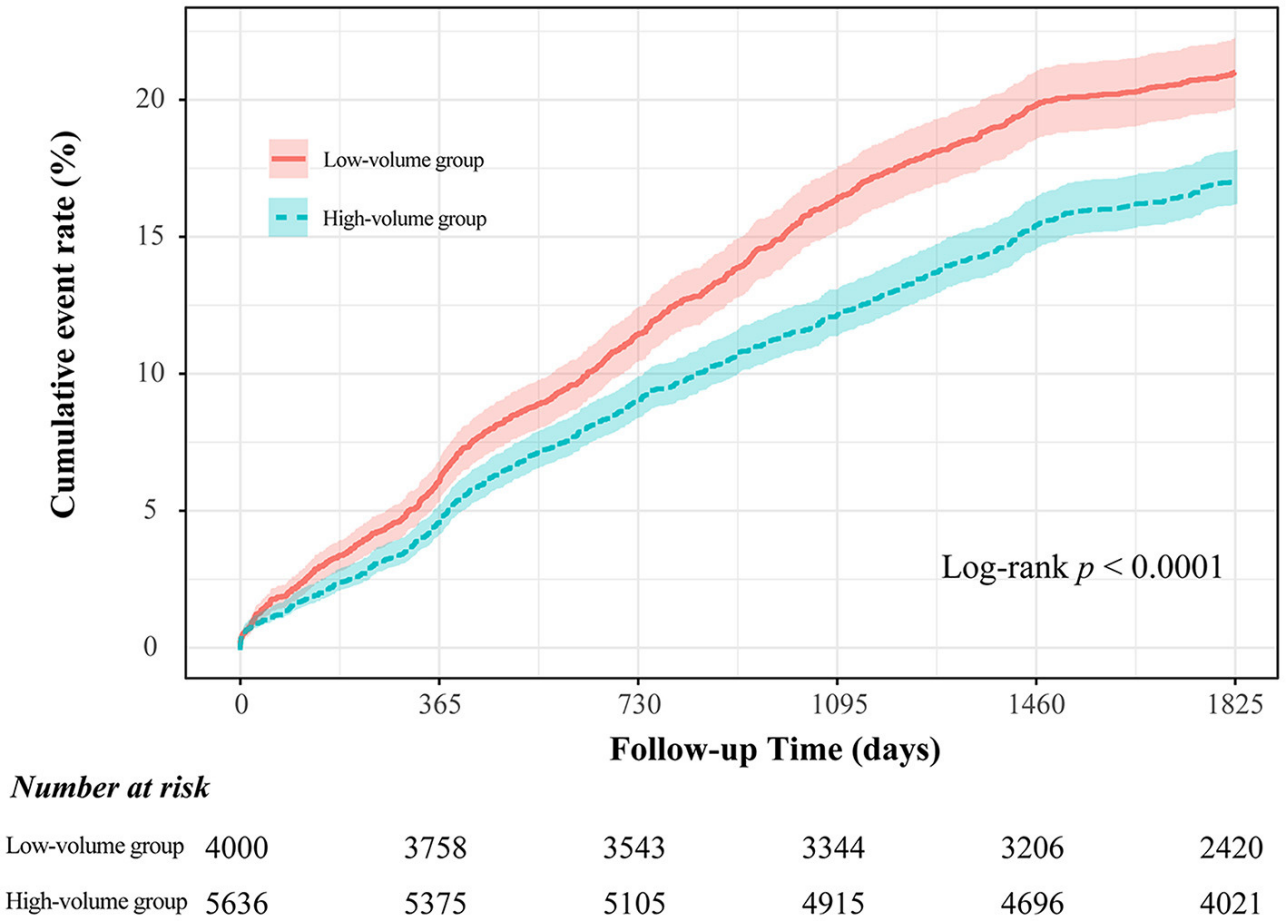
Time-to-event curves for the MACE through 5-year follow-up in the full cohort.

Based on the cut-off value derived from the restricted cubic spline model, patients were categorized into the high-volume group (exercise volume ≥ 6) and the low-volume group (exercise volume < 6) (Supplementary eFigure 3). The Kaplan–Meier curves for both cohorts were shown in Figure 6. The results showed that the cumulative incidence of MACE in the high-volume group was lower than that in the low-volume group (log-rank *P* < 0.0001).

## Discussion

In this cohort of patients with ACS, we developed and validated a simple-to-use nomogram model for predicting 1-, 3-, and 5-year MACE risk. This nomogram model contained an extensive set of clinical risk factors and exercise-related indicators which were considered protective factors for coronary heart disease (15, 16, 26). To our knowledge, this nomogram was the first clinical prediction model incorporating daily exercise to predict the short-, medium-, and long-term risk of MACE among patients with ACS. This prediction model performed well and helped to guide clinical decision-making and provide accurate predictions for ACS patients. In addition, the positive effect of exercise for such patients improved both the physicians' and patients' understanding of treatment and compliance with exercise training, which has great significance for reducing the MACE risk.

Using the multivariable regression analysis of MACE incidence among ACS patients, the 15 most important factors contained most of the prognostic information and were incorporated into the nomogram. Exercise, as a protective factor, has a great impact on the prognosis of ACS patients and decreases the incidence of MACE (17). A meta-analysis demonstrated that the incidence of MACE was significantly lower in the CR group compared with the non-CR group (RR, 0.49; 95%CI, 0.44–0.55; *P* < 0.05) (26). Doimo et al. (27) found that the exercise-based CR was an independent predictor for low occurrence of the composite outcome of hospitalizations for cardiovascular causes and cardiovascular mortality (HR, 0.58; 95%CI, 0.43–0.77; *P* < 0.001). As for patients with AMI, Pouche et al. (28) found that the CR program was significantly associated with the 5-year mortality among patients with AMI (HR, 0.64; 95%CI, 0.60–0.96). Moreover, this study also demonstrated that association was more obvious in patients with non-ST segment elevation myocardial infarction (NSTEMI) (HR, 0.63; 95%CI, 0.46–0.88). In addition, in a cohort of patients with myocardial infarction in the absence of obstructive coronary artery disease (MINOCA), exercise-based CR was also associated with a significantly low incidence of all-cause mortality (HR, 0.483; 95%CI, 0.279–0.818; *P* < 0.01) and MACE (HR, 0.574; 95%CI, 0.403–0.827; *P* < 0.001) (29). The potential mechanism for exercise to reduce the incidence of MACE lay in the improvement of myocardial oxygen supply, cardiac systolic function, cardiopulmonary fitness, and endothelial function, the regulation of autonomic nerves, coagulation factors, and inflammatory markers, and the development of coronary collateral vessels (17, 30–35). The exercise-based CR also exerted hypoglycemic, hypolipidemic, hypotensive and any other positive effects to reduce the risk factors related to coronary heart disease (36–39). These studies all suggested that exercise-based CR reduced the occurrence of MACE among patients with the entire spectrum of ACS. However, to our knowledge, there was no prediction model to predict MACE incorporating exercise. Therefore, the exercise intensity and exercise duration were incorporated into the model, which as core contents of exercise, could better assess patients' functional status and exercise compliance.

In addition, we noted that risk factors that affected the prognosis also included age, previous MI, previous PCI, systolic pressure, NT-proBNP, HDL, serum creatinine, LVEDD, Killip class, SYNTAX score, LAD stenosis, LCX stenosis, RCA stenosis. Consistent with the previous study, age is an independent predictor of adverse cardiovascular events (40, 41). Previous medical history, such as previous MI, and previous PCI was related to the ischemia risk of ACS patients, which predicted the occurrence of adverse events in such patients. In addition, several studies have demonstrated that systolic pressure, serum creatinine, and HDL played an important role in predicting the incidence of cardiovascular events (42, 43). NT-proBNP was a sensitive indicator for evaluating the cardiac function of patients, which predicted the mortality of patients with ACS (44, 45). Similarly, the LVEDD was also an important indicator reflecting the functional load of the cardiac pump, which also affected the prognosis of ACS patients (46). The Killip class and SYNTAX score reflected the cardiac function and complexity of coronary lesions, respectively, which were the determinants of the severity of ACS and also played an important role in predicting adverse outcomes (43, 47). As for ACS patients with the muti-vessel disease, Iqbal et al. (48) has demonstrated that the untreated proximal LAD (HR, 1.23; 95%CI, 1.06–1.51; *P* = 0.045) and RCA (HR, 1.36; 95%CI, 1.08–1.65; *P* = 0.007) were both associated with the increased mortality. Moreover, the TIMI risk score and the CADILLAC risk score indicated that prior coronary stenosis of 50% or more and three-vessel disease were both independent predictors of cardiovascular adverse events (10, 11). To overcome the limitation of a single predictor and incorporate as many predictors of different types as possible, we established the nomogram model based on all of the above variables.

To our knowledge, the TIMI risk score was the first internationally recognized scale, which was established to predict the 14-day incidence of the composite outcome of all-cause death, myocardial infarction, and severe recurrent ischemia requiring urgent revascularization for patients with unstable angina or NSTEMI (10). As for patients with ACS, the GRACE risk score has been established to predict the mortality risk during hospitalization and at 6 months (5). While among patients with AMI after PCI, both the PAMI risk score and CADILLAC risk score were established to predict the 30-day and 1-year mortality risk (8, 11). By comparing these risk scores, several studies have demonstrated the superiority of the CADILLAC risk score in predicting the 30-day and 1-year mortality risk, which was explained by the inclusion of LVEF and three-vessel disease in the risk score (49, 50). Our nomogram model has included all these variables except the postprocedural TIMI flow grade and ST-segment deviation, so that it made a good prediction for the MACE risk among ACS patients. Moreover, our nomogram, different from the previous risk scores, was developed with the large sample size, which was used to better predict the MACE risk compared with the previous risk scores. In addition, the long follow-up period enhanced the accuracy of model, which therefore was a major strength in comparison with the previous recognized risk scores.

In the present study, the C-index derived from the nomogram model was 0.645 in the derivation cohort and was confirmed to be 0.640 in the validation cohort as well, indicating the moderate discrimination of the models. The calibration plots and DCA curves analysis were also performed well, showing the credibility and broad applicability of our model in clinical practice. Compared with the traditional models, our nomogram model has several strengths. On the one hand, our current model was the first to incorporate exercise-related indicators to predict long-term MACE risk. On the other hand, our model helped to facilitate risk stratification, highlighted the importance of exercise among ACS patients, and guided the implementation of exercise-based CR in clinical practice as early as possible.

### Limitations

To date, our study was the first to evaluate the importance of exercise in ACS patients, however, there were still some limitations of this study. First, our study was a single-center retrospective study, which resulted in selection bias. So our future research will include patients from other areas of China to further validate our model. Second, the present study lacked external validation and didn't compare our model with other international models in the same cohort. Third, some potential variables were not involved in our model, such as the indices of cardiopulmonary exercise testing (CPET) and PCI-related parameters. However, our study population was ACS patients undergoing coronary angiography and the inclusion criteria were not limited to cases with CPET and PCI. Finally, the role of exercise volume including exercise intensity and cumulative time was not consistent during the whole process of study. As a result, the exercise volume was defined as the average level after collecting the related follow-up information.

## Conclusions

This study developed and validated a robust model incorporating the already known factors and daily exercise to predict 1-, 3-, and 5-year incidence of MACE among patients with ACS. This nomogram was well established to provide a simple-to-use method for ACS patients to accurately help high-risk patient identification and clinical decision-making. Additionally, we provided new insight into the important role of exercise and encouraged the physicians to widen the usage of exercise-based cardiac rehabilitation in ACS patients as early as possible.

## Data availability statement

The raw data supporting the conclusions of this article will be made available by the authors, without undue reservation. Further inquiries can be directed to the corresponding authors.

## Ethics statement

The studies involving human participants were reviewed and approved by the institutional Ethics Committee of the General Hospital of Northern Theater Command. Written informed consent for participation was not required for this study in accordance with the national legislation and the institutional requirements.

## Author contributions

Y-LH conceived the idea. Y-LH and Q-YZ designed the trial. QH, QR, and Y-SL performed the study. QR and Y-SL collected the data. P-XL checked the data. Y-CL and JZ scrubbed data and maintained research data. QH and P-XL performed the data analysis. QH wrote the manuscript. Q-YZ revised the paper. All authors contributed to the article and approved the submitted version.
