# Supplementary material for: Daily exercise improves the long-term prognosis of patients with acute coronary syndrome
Source: Front Public Health. 2023 Mar 15;11:1126413. doi: 10.3389/fpubh.2023.1126413 (PMC10050345; doi:10.3389/fpubh.2023.1126413)
Supplement: Supplementary file 1 [file Table_1.DOCX]

Supplementary Material

Daily exercise improves the long-term prognosis of patients with acute coronary syndrome

**Qiang Hu, Peng-Xiao Li, Yu-Shan Li, Qiang Ren, Jian Zhang, Yan-Chun Liang, Quan-Yu Zhang, Ya-Ling Han**

*** Correspondence:** Ya-Ling Han: [hanyaling@163.net](mailto:hanyaling@163.net); Quan-Yu Zhang: [zqyfmmu@foxmail.com](mailto:zqyfmmu@foxmail.com)

# Supplementary Figures and Tables

## Supplementary Figures


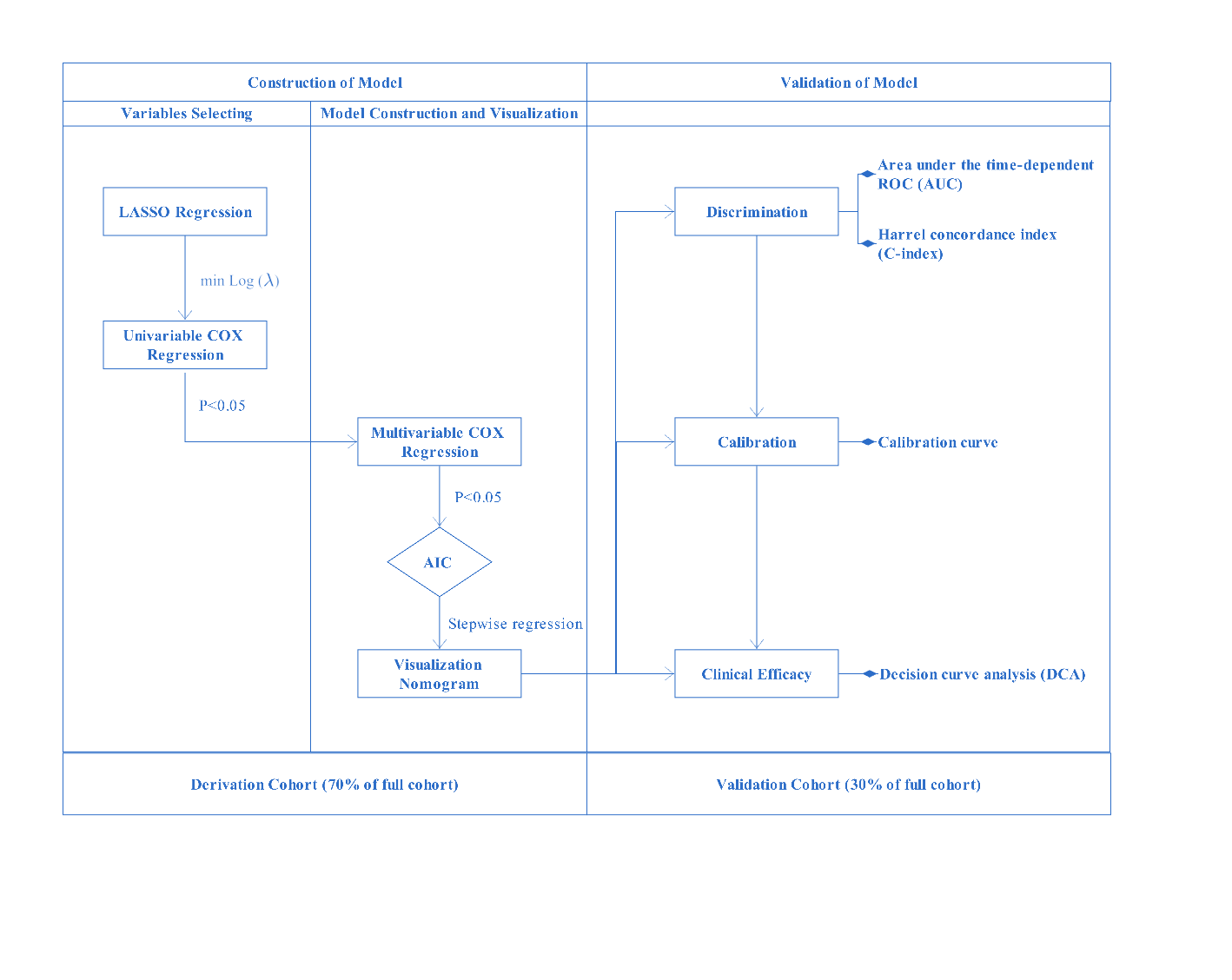


eFigure 1 Flow diagram of model construction and validation


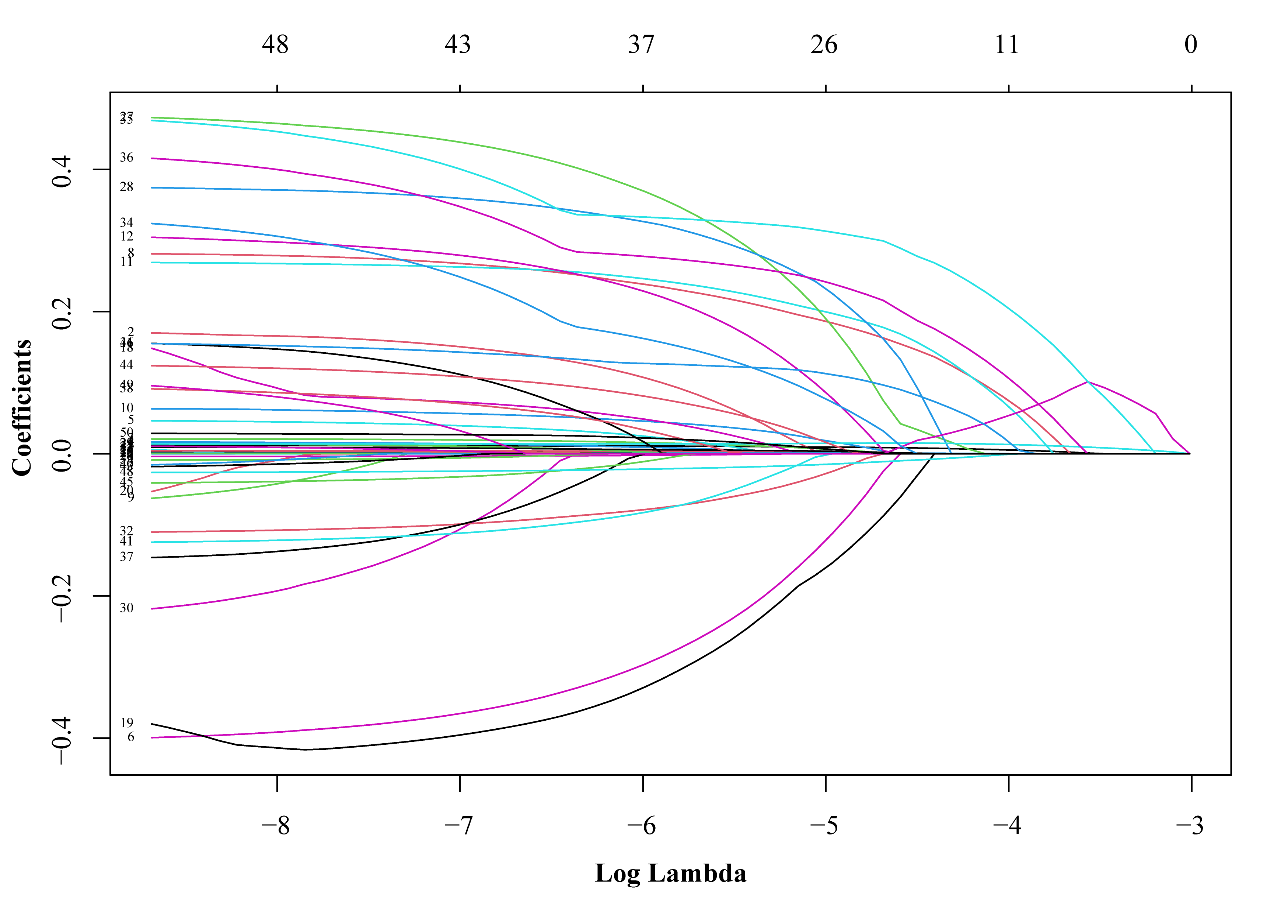


eFigure2 A


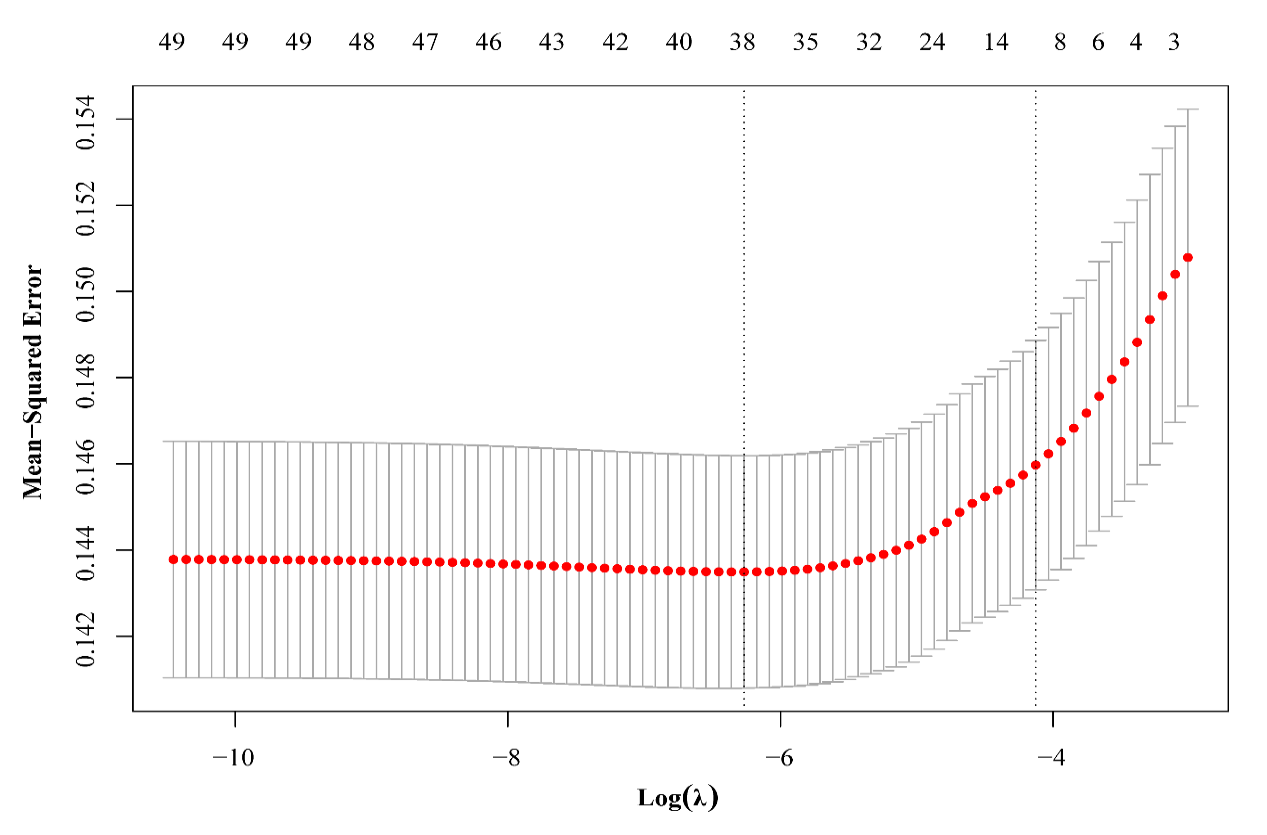


eFigure2 B

eFigure2 Variables selection using the least absolute shrinkage and selection operator (LASSO) binary logistic regression model. (A) LASSO coefficient profiles of the 50 baseline variables. (B) Tuning parameter (λ) selection in the LASSO model used 10-fold cross-validation via minimum criteria.


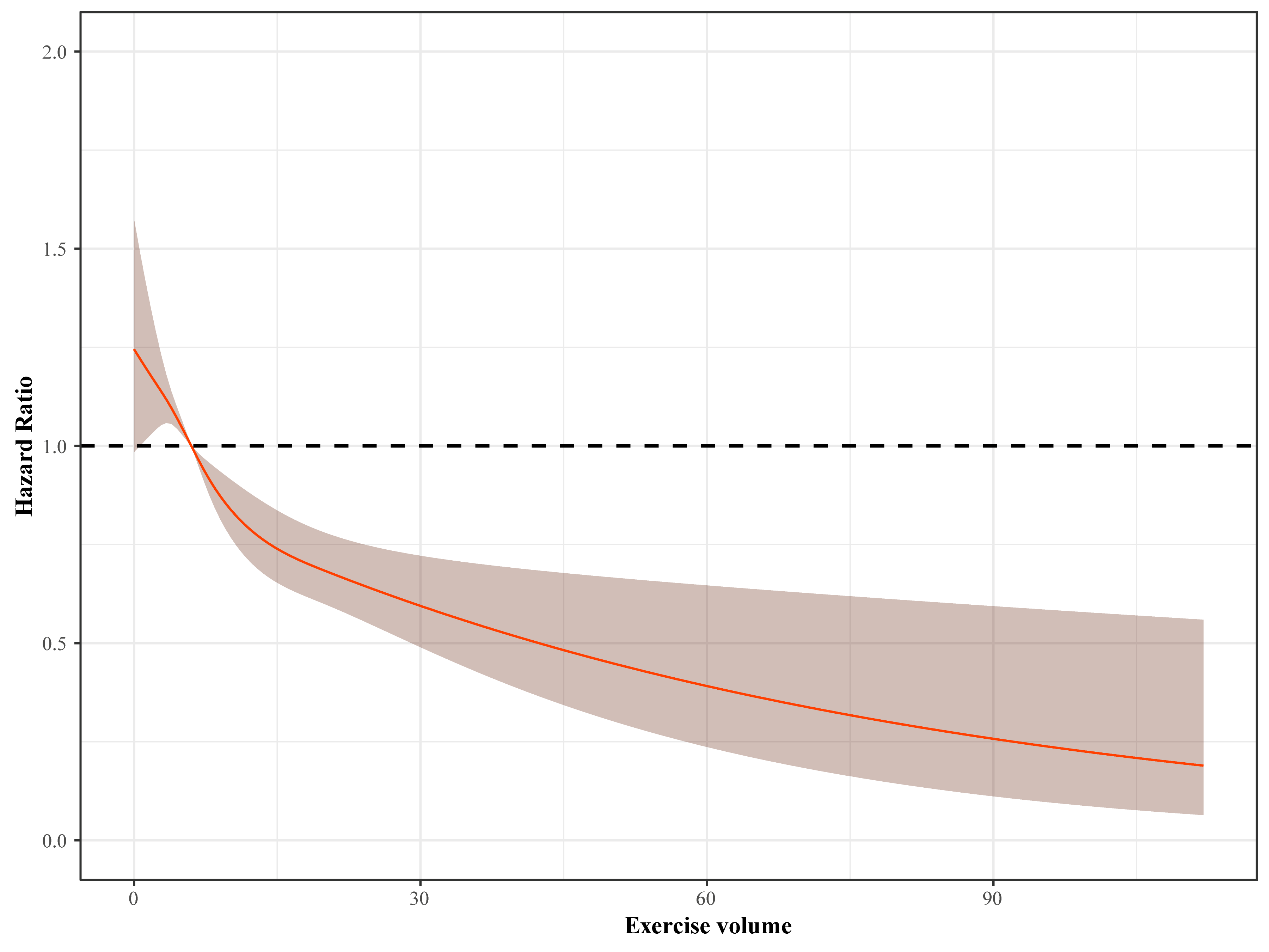


eFigure 3 Association of exercise volume with MACE in patients with ACS

## Supplementary Tables

| MET | Daily life | Interests and hobbies | Sports | Work |
| --- | --- | --- | --- | --- |
| 1-2 | Having a meal, face washing, sewing, driving a car | Listening to radio, reading, watching television, playing cards, weiqi, Chinese chess | A slow walk (1.6km/h) | Education, desk work |
| 2-3 | Stand in bus,  cooking,  washing small pieces of clothing,  wiping the floor (with a mop) | Bowling ,  gardening ,  playing golf (with the Cardinals), | Flat walking (3.2 km/h), strolling up to the second floor) | Doorkeeper,  administrator,  Instrument player |
| 3-4 | Taking a shower,  wiping the windows,  cooking,  making the bed,  walking with 10kg luggage,  sweeping,  wiping the floor kneeling | Doing radio gymnastics,  fishing,  playing Badminton (non-competitive),  Playing golf | A slightly brisk walk (4.8km/h) | Mechanical assembly agent,  trucker ,  taxi driver,  welder |
| 4-5 | Holding 10kg luggage on foot,  sweeping,  sexual intercourse, taking a bath, weeding slowly | Making ceramics,  dancing,  playing table tennis,  playing tennis,  catching a baseball,  playing golf | Walking fast (5.6km/h) | Fitter,  tile worker,  wallpaper,  light Carpenter |
| 5-6 | Taking 10kg luggage with one hand,  walking downhill,  loosening the soil with a spade | Stream Fishing,  skating | Scurrying (6.5 km/h) | Carpenter ,  farming |
| 6-7 | Digging earth,  snow cleaning | Aerobics,  recreational skiing (4km/h) | Jogging (4-5 km/h) | Road repair work,  cement workers,  logging workers |
| 7-8 |  | Swimming,  climbing,  skiing,  aerobics | Jogging (8.0 km/h) | Grazing |
| 8- | Climbing >10 floors in a row | Rope skipping,  all kinds of competitive sports | Jogging (8.9 km/h) | Boiler fireman |

eTable 1. Met equivalents of physical activity

eTable 2 Baseline demographics and clinical characteristics of patients in the derivation cohort and validation cohort

| **Characteristic** | **Derivation cohort (n=6745)** | **Validation cohort (n=2891)** | **P** |
| --- | --- | --- | --- |
| Age | 60.36±10.36 | 60.29±10.37 | 0.692 |
| Male | 5062(75.05%) | 2173(75.16%) | 0.904 |
| Body mass index | 24.33±3.09 | 24.36±3.11 | 0.711 |
| Smoking status |  |  |  |
| No smoke | 2803(41.56%) | 1233(42.65%) | 0.529 |
| Current smoker | 2931(43.45%) | 1244(43.03%) |  |
| Previous smoker | 1011(14.99%) | 414(14.32%) |  |
| Drinking status |  |  |  |
| No drink | 4999(74.11%) | 2210(76.44%) | 0.051 |
| Current drinker | 1376(20.40%) | 532(18.40%) |  |
| Previous drinker | 370(5.49%) | 149(5.15%) |  |
| Diagnosis |  |  |  |
| UA | 4612(68.38%) | 1936(66.97%) | 0.347 |
| NSTEMI | 879(13.03%) | 403(13.94%) |  |
| STEMI | 1254(18.59%) | 552(19.09%) |  |
| Hypertension | 4317(64.00%) | 1838(63.58%) | 0.690 |
| Previous MI | 1213(17.98%) | 529(18.30%) | 0.713 |
| Chronic kidney disease | 66(0.98%) | 29(1.00%) | 0.911 |
| Diabetes | 1793(26.58%) | 776(26.84%) | 0.792 |
| Previous PCI | 1280(18.98%) | 533(18.44%) | 0.534 |
| Stroke | 296(4.39%) | 147(5.08%) | 0.135 |
| Heart rate | 76.73±13.56 | 76.67±13.68 | 0.702 |
| Systolic pressure | 136.05±21.13 | 135.04±22.02 | 0.020 |
| Diastolic pressure | 78.46±12.44 | 78.05±12.83 | 0.117 |
| NT-proBNP | 201.90(70.55,721.80) | 205.40(68.34,772.85) | 0.781 |
| Triglyceride | 1.84±1.48 | 1.85±1.53 | 0.910 |
| LDL | 2.36±0.82 | 2.35±0.78 | 0.793 |
| HDL | 0.92±0.21 | 0.92±0.22 | 0.059 |
| Cholesterol | 4.16±1.14 | 4.15±1.09 | 0.992 |
| cTnT | 0.02(0.01,0.27) | 0.02(0.01,0.28) | 0.864 |
| Serum creatinine | 70.47(60.58,81.68) | 70.43(60.20,81.56) | 0.568 |
| CKMB | 12.00(10.00,18.00) | 12.00(10.00,18.00) | 0.788 |
| Hemoglobin | 136.53±15.20 | 136.32±15.21 | 0.911 |
| LVEDD | 49.63±5.99 | 49.84±6.16 | 0.419 |
| LVEF | 58.97±8.46 | 58.87±8.58 | 0.791 |
| Killip class |  |  |  |
| 0 | 4571(67.77%) | 1917(66.31%) | 0.558 |
| I | 1935(28.69%) | 875(30.27%) |  |
| II | 185(2.74%) | 73(2.53%) |  |
| III | 24(0.36%) | 11(0.38%) |  |
| IV | 30(0.44%) | 15(0.52%) |  |
| IABP | 71(1.05%) | 44(1.52%) | 0.052 |
| SYNTAX score | 13.96±8.85 | 14.07±9.23 | 0.785 |
| Three-vessel disease | 2578(38.22%) | 1085(37.53%) | 0.522 |
| Chronic total occlusion | 57(0.85%) | 13(0.45%) | 0.036 |
| Arterial access site |  |  |  |
| Femoral | 420(6.23%) | 169(5.85%) | 0.474 |
| Radial | 6325(93.77%) | 2722(94.15%) |  |
| Contrast | 100.00(40.00,180.00) | 100.00(40.00,180.00) | 0.426 |
| LAD stenosis (≥50%) | 5658(83.88%) | 2428(83.98%) | 0.902 |
| LCX stenosis (≥50%) | 3959(58.70%) | 1695(58.63%) | 0.953 |
| RCA stenosis (≥50%) | 4342(64.37%) | 1829(63.27%) | 0.299 |
| Number of stents | 1.38±0.88 | 1.39±0.88 | 0.678 |
| Aspirin | 6674(98.95%) | 2857(98.82%) | 0.593 |
| Clopidogrel | 5943(88.11%) | 2504(86.61%) | 0.041 |
| Ticagrelor | 1608(23.84%) | 745(25.77%) | 0.043 |
| Statin | 6555(97.18%) | 2823(97.65%) | 0.195 |
| Beta blocker | 4817(71.42%) | 2048(70.84%) | 0.567 |
| Calcium channel blocker | 1894(28.08%) | 833(28.81%) | 0.464 |
| Nitrate | 4449(65.96%) | 1842(63.71%) | 0.034 |
| Proton-pump inhibitor | 4511(66.88%) | 1915(66.24%) | 0.542 |
| ACEI/ARB | 4035(59.82%) | 1725(59.67%) | 0.888 |
| Diuretics | 1119(16.59%) | 514(17.78%) | 0.154 |
| Exercise intensity ^a^ |  |  |  |
| Mild | 710(10.53%) | 305(10.55%) | 0.844 |
| Moderate | 6008(89.07%) | 2572(88.97%) |  |
| High | 27(0.40%) | 14(0.48%) |  |
| Cumulative time ^b^ | 3.00(2.00,6.00) | 3.00(2.00,6.00) | 0.657 |
| Exercise type |  |  |  |
| No exercise | 22(0.33%) | 10(0.35%) | 0.718 |
| Walking | 6700(99.33%) | 2871(99.31%) |  |
| Riding | 5(0.07%) | 4(0.14%) |  |
| Running or Swimming | 18(0.27%) | 6(0.21%) |  |

Abbreviation: UA, unstable angina; NSTEMI, non-ST segment elevation myocardial infarction; STEMI, ST-segment elevation myocardial infarction; MI, myocardial infarction; PCI, percutaneous coronary intervention; NT-proBNP, N-terminal Pro-B-type natriuretic peptide; LDL, low density lipoprotein cholesterol; HDL, high density lipoprotein cholesterol; cTnT, troponinT; CKMB, creatine kinase isoenzyme; LVEDD, left ventricular end-diastolic diameter; LVEF, left ventricular ejection fraction; IABP, intra-aortic balloon counterpulsation; SYNTAX score, the Synergy between Percutaneous Coronary Intervention with Taxus and Cardiac Surgery score; LAD, left anterior descending; LCX, circumflex; RCA, right coronary artery; ACEI, angiotensin converting enzyme inhibitors; ARB, angiotensin receptor blockers.

^a^ Physical activity was categorized into mild (<3 METs), moderate (3 to 6 METs) and high intensity (>6 METs).

^b^ Cumulative exercise time was defined as the cumulative time of walking, light household activities, and exercise in a week.
